# Philadelphia Correspondence

**Published:** 1868-11-01

**Authors:** E. R. Hutchins

**Affiliations:** Philadelphia


### *PHILADELPHIA CORRESPONDENCE.*

---

PHILADELPHIA, *Oct.* 19*th*, 1868.

*Editor* CHICAGO MEDICAL JOURNAL:

As in other branches of medicine and surgery, improvements, constant and important, have been rapidly made, so in the treatment of *uterine* diseases, both its surgical and its medical departments have progressed and improved most astonishingly. Whereas, not many years ago, all uterine diseases were placed under the head of "spine diseases," or "inward complaints," and the patients were drugged and dosed, and left in a condition worse than their previous one, and it was only now and then that a speculum, or a vaginal or uterine examination, even by touch, was thought of. How widely different is it now! A medical man's library is incomplete without the modern works on this class of diseases. How the writings of Bennet and Linus, Bedford, Hewitt, and Simpson, and others, enhance the value of one's medical shelves now! Those who, like Buck, of whom I have before spoken, in that in which he has ever been a specialist—surgery—are of high order and merit, but who, like him, have too contracted views of other special diseases to see their importance, and who, probably, have made but a very limited observation among these diseases, pronounce *all* who engage in this class of practice, "*enthusiasts*" and "*hobbyists!*" Heaven knows, the mothers and wives of this and every country, would be far better, had many of them placed themselves in the hands of *such enthusiasts* long ago. Take, for instance, that class of uterine disorders so common among women — ulceration of the os uteri. Pointed out by its universal and constitutional symptoms — pain in lumbo-sacral region, constipated bowels generally, pain on top of head, etc.; a class of symptoms formerly, and even now, far too commonly calling in aid revulsives of all characters, and *alteratives;*—how simple is its cure! Statistics are the best proofs of any matter. I find I have recorded in my notes, the histories of one hundred and ninety-one cases of ulcerated os uteri. These, I have gathered from clinical and private practice. Many of them, indeed, the larger number, were those of a tedious, chronic character, the subjects having suffered for a period of years, and many of them having been under *constant* use of medicine internally. Of these, *every one was cured* by local interference. In one hundred and two cases, *Arg. nit.* was used; in forty cases, Monsell's solution was applied; in twenty-three, *Sulphate of Copper;* and in the remaining fourteen, *Tinc. Iodine.* The number of visits ranged from two to sixteen in each case, these being the minimum and maximum number. Is not this array of facts *alone* enough to induce practitioners more generally to adopt these means of cure?

Take, again, the subject of inflamed uteri—a disease pointed out by constitutional symptoms similar to the above, together with continued constipated bowels. An examination *per vagina* will reveal an angry, red uterus, hypertrophied, not unfrequently, to a very great extent. Among my notes, I have the history of ninety-seven cases of this character, of a duration ranging from three months, to a period not less than fifteen years. Some of the subjects are absolute burdens to themselves. Of these, eighty-three were *completely cured;* while the remaining fourteen were more or less relieved. The treatment was by means of *Arg. nit.*, *Caustic Potassa*, and sponge-tents. And in discussing this portion of my subject, I shall close this letter.

In the treatment of this form of uterine disease, the cure is very commonly attained by the first-named drug, *Arg. nit.;* but the objection is very properly advanced that it is slow in its action, and this objection is a valid one. I find, while the same patients would not object to taking medicine internally, from month to month, they object decidedly to being operated on for a long time; and the use of *Nitrate of Silver* necessitates this, often, though a deep and large eschar is made on one or both lips of the uterus. To remedy the difficulty, *Caustic Potassa* has been used; and this has a very decided objection, in its great escharotic power. I have used this drug in two cases well adapted for its use, and in both cases have been disappointed. The result, though *sooner* gained than by the use of *Arg. Nit.* is not of enough increased proportionate value to reward one for the great pains required in its use. Its corrosive effect is so decided and rapid, that it requires the most careful attention, with the liberal use of *Acetic acid*, and no little time. The third and last means I have mentioned, I can not commend in too high terms. I have, prior to this letter, cited several cases illustrative of its value, and I hope ere long to publish a monogram, giving concisely, and yet fully, the histories of the numerous cases which have fallen under my notice, as affording indubitable evidence of the value of the tent as a means of cure. A sponge tent, suitable for every purpose, is readily made by any physician, and can easily be formed into various sizes. I have seen it used (in my own and clinical practice) in sixty-four cases of hypertrophied uteri, and have yet to see a single case where it fails in its use. It seems to melt down a hypertrophy at times almost like magic. A tent is introduced through the internal os, up the entire uterine canal, and allowed to remain twenty-four hours. It is then removed, and a larger one introduced, and it will rarely happen that the hypertrophy is not decidedly decreased by the forty-eighth hour. These tents, to be sure, are not without their evil effects. Like *Calomel*, they produce serious mischief some times. Acute metritis will occasionally, but rarely, follow. If it is necessary to use a disinfectant, the tent can readily be medicated by *Per-manganate of potash* or *Carbolic acid.* I shall resume this subject in my next. Yours,

E. R. HUTCHINS.

